# A plant-based diet index to study the relation between diet and disease risk among adults: a narrative review

**DOI:** 10.1016/j.jnha.2024.100272

**Published:** 2024-05-29

**Authors:** Kerstin A. Schorr, Venetka Agayn, Lisette C.P.G.M. de Groot, P. Eline Slagboom, Marian Beekman

**Affiliations:** a Innoso BV, Den Haag, The Neterhlands; b Leiden University Medical Center, Leiden, The Netherlands; c Wageningen University and Research, Wageningen, The Netherlands

**Keywords:** Plant-based diet, Age-related diseases, Cardiovascular disease, Type 2 diabetes, Nutritional epidemiology

## Abstract

Plant-based diets (PBD) may offer various health benefits and contribute to a sustainable way of life, but, if not planned correctly, may also confer risks, e.g., by focusing on plant foods with low nutrient density, such as foods primarily consisting of refined carbohydrates. A plant-based diet index (PDI) differentiating between a healthful, unhealthful, and overall PBD, offers a promising approach to standardize and compare studies and integrate results. In this review we (1) summarize current evidence on the PDI and disease risk of relevance to public health, (2) discuss the methodology of the PDI and how it can be sensibly applied in further studies and (3) indicate areas with a lack of knowledge, such as vulnerable populations. In summary, our amalgamation shows, that adherence to a healthier plant-based diet is associated with an 8–68% lower risk for metabolic risk factors, diabetes, and cardiovascular disease, while adherence to an unhealthier plant-based diet is associated with a 10–63% higher risk. Although differences in calculation methods and underlying diet patterns between populations should be accounted for, the PDI can be a useful tool to assess adherence to different plant-based diet patterns and their association with health outcomes in cohort studies across cultures.

## 1 Introduction

Plant-based diets are generally defined as diets consisting only or mostly of plant foods. Accordingly, they can encompass a wide variety of diets, including vegan, vegetarian or even omnivorous diet with small amounts of animal foods, such as the Mediterranean diet [1,2]. Plant-based diets are recommended by dietary organizations worldwide [3,4], as they are an important tool to reach sustainability goals and may contribute to a lower incidence of multimorbidity and can therefore promote healthy ageing [[5], [6], [7], [8], [9], [10]].

However, the term plant-based is not clearly defined and is for example also used to describe diets, such as the Mediterranean diet which focus on specific foods that are not widely consumed in different parts of the world. The lack of clear definition of the term plant-based, has in the past caused ambiguity among researchers as to what types of diets fall under the term plant-based [11]. Applying an objective quantification of the amount of plant foods consumed in a diet is vital and different dietary indices have been developed for this purpose. However, the large number of dietary indices and the lack of a clear definition for the term plant-based make comparison between studies difficult, leading to uncertainty on the adequate plant-based diet composition [11].

Differences within the group of plant foods exist in the degree to which a food is considered healthful or associated with adverse health outcomes [[12], [13], [14]]. A plant-based diet index developed by Satija et al. aims to address this by combining the quantification of plant-based diet adherence with an evaluation on the healthfulness of the diet. It does so by differentiating between a healthful (hPDI) and unhealthful (uPDI) plant-based diet index. The latter predominantly consists of foods previously associated with an increased risk for cardiometabolic diseases, such as sugary drinks and foods high in refined carbohydrates whereas the former is mainly composed by whole plant foods, such as fruits and vegetables, wholegrains, or legumes [15]. The index is calculated based on food frequency questionnaire (FFQ) data by grouping consumed items into predetermined food groups based on epidemiological evidence and dividing the intake per food group in quintiles whereby each quintile is assigned a score from 1 to 5. For the overall PDI all plant-food groups are scored positively, meaning participants with intake in the highest quintile receive a score of 5, while animal food groups are scored inversely, meaning intake above the highest quintile results in a score of 1. For the hPDI healthy plant-based foods are scored positively, while all remaining food groups are scored inversely, whereas the opposite scoring is applied to retrieve the uPDI (Table S1). Higher scores indicate higher adherence to the respective diet pattern [15]. Because the plant-based diet index is suitable to overcome difference in composition and cultures, we summarize what is known on the relation between the plant-based diet index with age-related diseases. The validity and reliability of the PDI have been confirmed by previous research [16], rendering it a promising method to quantify plant-based diet adherence and a valuable tool to study plant-based diets in various compositions and compare studies across cultures.

Since its publication, the plant-based diet index by Satija et al. has been applied in a variety of studies, yet its strengths and weaknesses have not been discussed. The aim of this review is therefore (1) to give an overview over relations on the plant-based diet index and relevant health outcomes among adults and (2) to consider its methodological strengths and weaknesses and (3) give recommendations for future research by identifying knowledge gaps and discussing future applications of the PDI.

## 2 Methods

For the purpose of this narrative review, we focus on publications associating the PDI by Satija et al. to non-communicable, metabolic diseases and health outcomes with high prevalence or relevance for public health and healthy ageing [[17], [18], [19]]. Further, we are interested in effect modifications by age and sex.

We conducted a search in PubMed and results were filtered by year, including papers published between June 2016 and 2023 (Mat S1). This was chosen since the plant-based diet index was first published in the year 2016. Included were studies that applied the plant-based diet index (or a modification of which), and that assessed the association with one of the above-mentioned health outcomes and that were published until end of March 2023. We included studies in adults of all ages. Due to the nature of the index as a tool for epidemiological studies, we focused on cross-sectional and prospective case-control or cohort studies.

## 3 Results

The search subsequently yielded 730 results, of which titles and abstracts were screened for whether they applied the plant-based diet index and looked at an outcome of interest. Subsequently, 50 original research articles of population-based studies were considered for inclusion in this publication. Eleven studies were identified through other sources, such as reference lists from other publications and free search in Google Scholar using the same search terms, resulting in 61 publications of mostly cross-sectional and prospective cohort studies in total to be summarized in this review (Table 1, Table 2, Table 3, Table 4, Table 5, Fig. S1). Out of the identified publications, 49 focused on outcomes of metabolic and cardiovascular health, for that reason those outcomes were the primary focus of this review, whereas 12 articles studied the gut microbiome or cognitive impairment. The mean age ranged from 36 to 81 years, with participants from at least 23 different countries.

**Table 1:** Overview over studies assessing the association between the plant-based diet index and cardiovascular diseases.

Study			Population characteristics			
Author, year [ID]	Outcome, assessment method	Study design	N (% female)	Age in years	Country	Main findings
Baden,2021 [21]	Stroke n_total_ = 6241 n_ischemic_ = 3015 n_hemorrhagic_ = 853, medical records	Prospective	NHS: 73,890 (100%) NHSII: 92,352 (100%) HPFS: 43,266 (0%)	NHS: 51 ± 7 NHS II: 37 ± 5 HPFS: 54 ± 10	US	HR_total_ = 0.90 (0.83, 0.98) comparing extreme quintiles of PDI
Chen, 2022 [22]	Cardiovascular disease n_CVDevents_ = 232, self-reported	Prospective	10,293 (57.9%)	40.7 ± 0.4	US	RR_CVD_ = 0.74 (0.60, −0.93) per 1-SD increment of hPDI
Heianza, 2020 [23]	Cardiovascular disease n_CVDevents_ = 1812, medical/death registry	Prospective	156,148 (54.5%)	56 ± 8	UK	HR_CVD_ = 0.90 (0.84, 0.97) per 10-unit increment of hPDI, no interaction with GRS
Heianza, 2021 [24]	Cardiovascular disease n_CVDevents_ = 1033, medical/death registry	Prospective	121,799 (57.4%)	55.1 ± 7.9	UK	HR_MI_ = 0.54 (0.39, 0.74) among high GRS group for hPDI, p_interaction GRSXhPDI_<0.001 on BMI
Kim, 2019 [25]	Cardiovascular disease n_CVDevents_ = 4381, self-reported, hospital records	Prospective	12,168 (55.9%)	53.8 ± 5.7	US	HR_CVD_ = 0.84 (0.75, 0.92) for highest vs. lowest quintile of PDI
Kouvari, 2022 [26]	Cardiovascular disease risk n_CVDevents_ = 317, self-reported, death registries	Prospective	2020 (45.8%)	39.8 ± 10.9	Greece	HR_CVD_ = 0.32 (0.16, 0.63) for hPDI comparing extreme tertiles
Lazarova, 2022 [27]	Cardiovascular disease (CCHS 2004) n_events_ = 748, hospital and death records	Cross-sectional	CCHS 2004: 6771 (na)	na	Canada	No significant association with CVD risk
Satija, 2017 [28]	Cardiovascular disease n_CHD_ = 8631, medical and death records	Prospective	NHS: 73,710 (100%) NHSII: 92,329 (100%) HPFS: 43,259 (0%)	NHS: 50.0 ± 7.1 NHSII: 36.3 ± 4.6 HPFS: 53.3 ± 9.5	US	HR_CHD_ = 0.92 (0.83,1.01), 0.75 (0.68, 0.83) and 1.32 (1.20,1.46) comparing extreme deciles of PDI, hPDI and uPDI, respectively
Shan, 2020 [29]	Cardiovascular disease n_CHD_ = 18,092 n_stroke_ = 5687, medial and death records	Prospective	NHS: 74,930 (100%) NHSII: 90,864 (100%) HPFS: 43,339 (0%)	NHS: 50.2 ± 7.2 NHSII: 36.1 ± 4.7 HPFS: 53.2 ± 9.6	US	HR_CVD_ = 0.86 (0.82,0.89), HR_Stroke_ = 0.92 (0.85,1.00), HR_CHD_ = 0.84 (0.80,0.87) per 25-percentile higher hPDI
Thompson, 2023 [30]	Cardiovascular disease n_CVD_ = 6890 medical and death records	Prospective	126,394 (55.9%)	56.1 ± 7.8	UK	HR_CVD_ = 0.92 (0.86, 0.99), comparing extreme quartiles of hPDI
Weston, 2022 [31]	Cardiovascular disease _CVDevents_ = 293, Self-reported, hospital records	Prospective	3635 (64.3%)	53.8 ± 12.5	US	No significant associations

hPDI = healthful plant-based diet index, uPDI = unhealthful plant-based diet index, PDI = plant-based diet index, FFQ = Food Frequency Questionnaire, T2D = Type 2 Diabetes, WC = Waist Circumference, FBS = Fasting blood sugar, GRS = Genetic Risk Score, HPFS= Health Professional Follow-up Study, CCHS = Canadian Community Health Survey, UK = United Kingdom, FI = Finland, NZ = New Zealand, BG = Bulgaria, AU = Australia, US = United States, na = not available.

**Table 2:** Overview over studies assessing the association between the plant-based diet index and Type 2 Diabetes.

Study				Population characteristics			
1st author	Outcome, assessment method	Study design	N (% female)		Age in years	Country	Main findings
Bhupathiraju, 2022 [33]	Cardiometabolic risk factors, Blood draw, anthropometry	Cross-sectional Prospective	891 (47.2%) 735 (na)		55.2 ± 0.64	South Asia	Per 5-uni increase in PDI: β_fasting glucose_ = 1.03 ± 0.35, β _HOMA-IR_= −3.46 ± 1.65, Per 5-unit increase in hPDI: β_HbA1c_ = −0.43 ± 0.14,β _HOMA-IR_ = −4.02 ± 1.42, OR_T2D_ = 0.82 (0.67,1.00)
Chen, 2018 [34]	Type 2 Diabetes n_cases_ = 5207, self-reported	Prospective	45,411 (55%)		55.0 (45–74)	Singapore	HR_T2D_ = 0.83 (0.76,0.92) for PDI and 0.81 (0.75, 0.89) for hPDI for highest vs. lowest quintile
Chen, 2021 [35]	Type 2 Diabetes n_cases_ = 12,627, Questionnaire based on official criteria	Prospective	NHS: 76,530 (100%) NHSII: 81,569 (100%) HPFS: 34,468 (0%)		NHS: 58.1 ± 7.9 NHSII: 41.1 ± 5.4 HPFS: 57.5 ± 9.7	US	HR_T2D_ = 1.12 (1.05,1.20) for PDI and 1.23 (1.16, 1.31) for hPDI for largest decrease (>10%) vs. stable indices
Chen, 2018 [36]	Type 2 Diabetes n_cases_ = 642, blood measurements	Prospective	6798 (58.7%)		62.7 ± 7.8	Netherlands	HR_T2D_ = 0.87 (0.79,0.99), β_insulin resistance_= −0.05 (−0.06, −0.04) for PDI per 10-unit increase
Flores, 2021 [37]	Type 2 Diabetes n_cases_ = 134, blood measurement	Prospective	646 (72%)		55.5 ± 0.5	Puerto Rico	HR_T2D_ = 0.54 (0.31,0.94) for hPDI for comparing highest vs. lowest tertile
Goode, 2023 [38]	Insulin Sensitivity, blood measurements	Prospective	667 (50.2%)		31.5 ± 2.6	Australia	β_insulin-sensitivity_ = 0.11 (0.05, 0.17) between-person and 0.10 (0.04, 0.16) within-person effect for hPDI
Kim, 2022 [39]	Type 2 Diabetes n_cases_ = 977, blood measurements	Prospective	7363 (55%)		52 ± 8.5	South Korea	HR_T2D_ = 0.86 (0.77,0.95) for hPDI for comparing highest vs. lowest tertile
Laouali, 2021 [40]	Type 2 Diabetes n_T2D_ = 3292), self-reported	Prospective	74,552 (100%)		52.9 + 6.7	France	HR_T2D_ = 0.71 (0.63,0.79) for PDI and 0.74 (0.67, 0.83) for hPDI
Satija, 2016 [15]	Type 2 Diabetes n_cases_ = 16,162	Prospective	NHS: 69,949 (100%) NHSII: 90,239 (100%) HPFS: 40,539 (0%)		NHS: 50 ± 7 NHSII: 36 ± 5 HPFS: 53 ± 9	US	HR_T2D_ = 0.80 (0.74,0.87), 0.66 (0.61, 0.72), 1.16 (1.08,1.25) for PDI, hPDI and uPDI, respectively for comparing extreme deciles
Yang, 2021 [41]	Type 2 Diabetes n_cases_ = na, self-reported	Prospective	37,985 (60.7%)		55.7 ± 12.2	China	OR_T2D_ = 0.88 (0.79, 0.98) for PDI comparing extreme quartiles
Zhang, 2023 [42]	Type 2 Diabetes n_cases_ = 7654, blood measurements	Cross-sectional	50,694 (59.6%)		55.3 ± 9.7	China	OR_T2D_ = 0.83 (0.75,0.92) for high CVD risk population and 0.80 (0.74,0.87) for non-high CVD risk population comparing extreme quartiles

hPDI = healthful plant-based diet index, uPDI = unhealthful plant-based diet index, PDI = plant-based diet index, FFQ = Food Frequency Questionnaire, T2D = Type 2 Diabetes, WC = Waist Circumference, FBS = Fasting blood sugar, GRS = Genetic Risk Score, HPFS= Health Professional Follow-up Study, CCHS = Canadian Community Health Survey, UK = United Kingdom, FI = Finland, NZ = New Zealand, BG = Bulgaria, AU = Australia, US = United States, na = not available.

**Table 3:** Overview over studies assessing the association between the plant-based diet index and metabolic risk factors.

Study			Population characteristics			
1st author	Outcome, assessment method	Study design	N (% female)	Age in years	Country	Main findings
Kim, 2021 [43]	Hypertension n_events_ = 2244, measured, self-reported or diagnosed	Prospective	5639 (53.3%)	50.6 ± 8.5	South Korea	HR_Hypertension_ = 0.65 (0.57, 0.75) for hPDI and 1.44 (1.24, 1.67) for uPDI comparing extreme quintile
Laouali, 2021 [40]	Hypertension n_Hypertension_ = 12,504 self-reported	Prospective	74,552 (100%)	52.9 + 6.7	France	HR_Hypertension_ = 0.89 (0.44,0.94) for PDI, 0.83 (0.78, 0.88) for hPDI and 1.10 (1.04, 1.17) for uPDI comparing extreme quintiles
Lazarova, 2022 [27]	Obesity	Cross-sectional	CCHS 2004: 6771 (na)	na	Canada	OR_obesity_ = 1.63 (1.30, 2.05) for unhealthiest vs. healthiest quartile
Bhupathiraju, 2022 [33]	Cardiometabolic risk factors, Blood draw, anthropometry	Cross-sectional Prospective	891 (47.2%) 735 (na)	55.2 ± 0.64	South Asia	Per 5-unit increase in PDI: OR_obesity_ = 0.86 (0.77,0.97) β_LDL-C_ = −0.08 ± 0.02 Per 5-unit increase in hPDI: β_visceral fat_ = −2.55 ± 0.92,β _adiponectin_ = 2.32 ± 1.08 OR_obesity_ = 0.88 (0.80,0.97) β_LDL-C_ = −0.04 ± 0.02β _Adiponectin_ = 2.32 ± 1.08 Per 5-unit increase in uPDI: β_LDL-C_ = −0.04 ± 0.02
Amini, 2021 [44]	Metabolic Syndrome n_cases_ = 95, anthropometry, blood measurements	Cross-sectional	178 (71%)	67.0 ± 6.1	Iran	No significant association
Jafari 2023 [45]	Metabolic Syndrome n_cases_ = 607, anthropometry, blood measurements	Cross-sectional	2225 (46.7%)	45.6 ± 8.2	Iran	OR_metS_ = 0.67 (0.52, 0.86) for highest vs. lowest tertile of hPDI
Kim, 2020 [46]	Metabolic Syndrome n_cases_ = 2583, NCEP-ATP III classification, anthropometry, blood measurements	Prospective	5646 (48.3%)	51.0 ± 8.6	South Korea	OR_obesity_ = 1.23 (1.06, 1.42) for extreme quintiles of uPDI
Kim, 2021 [47]	Metabolic Syndrome n_cases_ = 3367, anthropometry, blood measurements	Prospective	14,450 (61.3%)	41.3 ± 0.4	South Korea	HR_obesity_ = 1.46 (1.25, 1.71) for extreme quintiles of uPDI
Asoudeh, 2023 [48]	Adiposity, anthropometry	Cross-sectional	6724 (57%)	36.8 ± 8.08	Iran	No significant associations
Baden, 2019 [49]	Adiposity-related biomarkers, blood measurements	Prospective	831 (100%)	45 ± 5	US	Per 10-point higher hPDI: Cross-sectional Leptin: −7.2% (−11.0, −3,1),Insulin: −10.0% (−14.2, −5.6)hsCRP: −13.6% (−20.5, −6.1) sOB-R: 1.9% (0.3,3.7) Adiponectin: 3.0% (0.4, 5.7) Longitudinal: Leptin: −7.7% (−13.6, −0.4)hsCRP: −17.8% (−26.3, −8.4) Per 10-point higher uPDI:
Chen, 2019 [50]	Adiposity, anthropometry	Prospective	9633 (58%)	64.2 ± 8.7	Netherlands	Per 10-unit higher PDI: β_BMI_ = −0.70 kg/m^2^ (−0.81, −0.59)β _WC_= −2.0 cm (−2.3, −1.7) β _FMI_= −0.66 kg/m^2^ (−0.80, −0.52)β _BF%_= −1.1 points (−1.3, −0.84)
Ratjen, 2020 [51]	Adipose tissue volume, MRI	Cross-sectional	578 (43%)	62 (55–71)	Germany	Per 10-unit higher hPDI −4.9% (−8.6, −2.0) visceral adipose tissue
Satija, 2019 [52]	Weight Change, Self-reported	Prospective	NHS: 46,790 (100%) NHSII: 59,217 (100%) HPFS: 20,975 (0%)	NHS: 52 ± 7.1 NHSII: 37 ± 4.4 HPFS: 50 ± 7.7	US	Per 1-SD increase in PDI 0.04 kg (0.05, 0.02) and in hPDI 0.68 kg (0.69, 0.66) less weight gain and 0.36 (0.34, 0.37) more weight gain for uPDI
Shahavandi, 2020 [53]	Adiposity, anthropometry	Cross-sectional	270 (56.3%)	36.5 ± 13	Iran	OR_visceralAdiposity_ = 5.7 (1.15, 28.10) for extreme deciles of uPDI
Waterplas, 2020 [54]	BMI, WC, blood lipids, anthropometry, blood measurements	Prospective	650 (51.1%)	46 ± 9.2	Belgium	β_BMI_ = 0.135 for increases in PDI
Zhu, 2021 [55]	Weight maintenance, cardiometabolic risk factors, DXA, blood measurements, anthropometry	Prospective	710 (69.2%)	57 (46−63)	FI, UK, BG, NZ, AU	Δbodyweight −0.25 (−0.48, −0.002) for PDI adherence
Lee, 2021 [56]	Dyslipidemia n_cases_ = 2995, blood measurements	Prospective	4507 (58.7%)	51.8 ± 8.9	South Korea	HR_dyslipidemia_ = 0.78 (0.69, 0.88) for PDI, 0.63 (0.56, 0.70) for hPDI and 1.48 (1.30,1.69) for uPDI when comparing extreme quintiles
Song, 2021 [57]	Dyslipidemia n_cases_ = 48,166, blood measurements	Prospective	147,945 (62.9%)	53.2 ± 8.2	South Korea	HR_dyslipidemia_ = 1.15 (1.11,1.20) for extreme quintiles of uPDI
Shin, 2021 [58]	Dyslipidemia n_cases_ = 6658	Cross-sectional	14,167 (61.8%)	40.8 ± 0.1	South Korea	OR_dyslipidemia_ = 1.22 (1.05, 1.41), OR_highTG_ = 1.48 (1.21, 1.81), OR_lowHDL_ = 1.16 (1.00,1.35) for extreme quintiles of uPDI
Wang, 2023 [59]	Dyslipidemia n_cases_ = 1501, blood measurements	Cross-sectional	4096 (55.1%)	51.23 ± 10.2	China	OR_dyslipidemia_ = 0.80 (0.66, 0.97) for PDI comparing quintile 4 vs. quintile 1 OR_lowHDL_ = 0.64 (0.49, 0.82) for PDI, 0.66 (0.50, 0.87) for hPDI, 1.35 (1.04, 1.74) for uPDI
Lotfi, 2022 [60]	Cardiometabolic risk factors, Anthropometry, blood measurements	Cross-sectional	3678 (na)	55.6 ± 7.9	Iran	OR_FBS_ = 0.42 (0.33, 0.53) for PDI, OR_totalChol_ = 0.80 (0.65, 0.98) for hPDI, OR_FBS_ = 1.23 (1.00, 1.53) and OR_totalChol_ = 1.23 (1.01,1.49), OR_FBS_ = 1.39 (1.13, 1.71) for uPDI,
Shirzadi, 2022 [61]	Cardiovascular risk factors, Anthropometry, blood measurements	Cross-sectional	371 (100%)	30.7 ± 6.9	Iran	Lower LDL-C in Tertile 3 vs. Tertile 1 of PDI (79.6 ± 14.4 vs. 83.0 ± 15.0, p = 0.021), Higher TG in Tertile 3 vs. Tertile 1 of uPDI (101.5 ± 56.6 vs. 97.7 ± 56.5)

hPDI = healthful plant-based diet index, uPDI = unhealthful plant-based diet index, PDI = plant-based diet index, FFQ = Food Frequency Questionnaire, T2D = Type 2 Diabetes, WC = Waist Circumference, FBS = Fasting blood sugar, GRS = Genetic Risk Score, HPFS= Health Professional Follow-up Study, CCHS = Canadian Community Health Survey, UK = United Kingdom, FI = Finland, NZ = New Zealand, BG = Bulgaria, AU = Australia, US = United States, na = not available.

**Table 4:** Overview over studies assessing the association between the plant-based diet index and mortality.

Study			Population characteristics			
Authors	Outcome, assessment method	Study design	n (% female)	Age in years	Country	Main findings
Kim, 2019 [25]	CVD- and all-cause mortality n_deaths_ = 5436 n_CVDdeaths_ = 1565, self-reported, hospital records	Prospective	12,168 (55.9%)	53.8 ± 5.7	US	HR_all-cause mortality_ = 0.75 (0.69, 0.82) for PDI and 0.89 (0.8, 0.98) for hPDI, HR_CVD-mortality_ = 0.81 (0.68, 0.97) for PDI and 0.68 (0.58, 0.80) for hPDI
Lazarova, 2022 [27]	Cardiovascular disease (CCHS 2004, n_events_ = 748), Hospital and death records	Cross-sectional	CCHS 2004: 6771 (na)	na	Canada	No significant association with CVD risk
Thompson, 2023 [30]	Mortality n_deaths_ = 5627 n_CVDdeaths_ = 698, medical and death records	Prospective	126,394 (55.9%)	56.1 ± 7.8	UK	HR_all-cause mortality_ = 0.84 (0.78, 0.91), comparing extreme quartiles of hPDI, HR_all-cause mortality_ = 1.23 (1.14, 1.32) comparing extreme quartiles of uPDI
Weston, 2022 [31]	All-cause mortality n_deaths_ = 597, Self-reported, hospital records	Prospective	3635 (64.3%)	53.8 ± 12.5	US	No significant associations
Baden, 2019 [68]	Total mortality n_deaths_ = 17,176 Cause-specific mortality n_CVDdeaths_ = 3918, death records, family reports	Prospective	NHS 49,407 (100%) HPFS 25,907 (0%)	NHS 63.7 HPFS 62.9	US	HR_all-cause mortality_ = 0.95 (0.90,1.00) for PDI, 0.90 (0.85,0.95) for hPDI and 1.12 (1.07,1.18) for uPDI, comparing greatest increase vs. stable diet scores HR_CVD-mortality_ = 0.93 (0.88, 0.99) for PDI, 0.91 (0.86, 0.96) for hPDI and 1.08 (1.02, 1.14) for uPDI per 10-point increase in diet index
Delgado-Velandia, 2022 [69]	All-cause mortality n_deaths_ = 699 CVD mortality n_CVDdeaths_ = 157, death records	Prospective	11,825 (54.4%)	47.0 ± 0.3	Spain	HR_all-cause mortality_ = 0.86 (0.74, 0.99) and HR_CVD-mortality_ = 0.63 (0.46, 0.85) per 10-unit increase in hPDI
Kim, 2018 [70]	Total mortality n_deaths_ = 2228, death records Cause-specific mortality n_CVDdeaths_ = 543	Prospective	11,879 (54%)	41.3 ± 0.6	US	HR_all-cause mortality_ = 0.95 (0.91, 0.98) per 10-unit increase in hPDI only in those with hPDI above median
Kim, 2021 [71]	Total mortality n_deaths_ = 3074 Cause-specific mortality n_CVDdeaths_ = 447, death records	Prospective	118,577 (65.1%)	52.7 ± 8.2	South Korea	HR_all-cause mortality_ = 0.76 (0.68, 0.85) for extreme quintiles of PDI HR_all-cause mortality_ = 1.30 (1.15, 1.48) for uPDI HR_CVD-mortality_ = 1.55 (1.08, 2.25) for extreme quintiles of uPDI
Li, 2021 [72]	Total mortality n_deaths_ = 4904 Cause-specific mortality n_CVDdeaths_ = 1029, death records	Prospective	40,074 (52%)	47.3 ± 19.4	US	HR_all-cause mortality_ = 0.80 (0.73, 0.89) for extreme quintiles of PDI and 0.86 (0.77, 0.95) for hPDI and 1.33 (1.19, 1.48) for uPDI HR_CVD-mortality_ = 1.42 (1.12, 1.79) for uPDI
Ratjen, 2021 [73]	All-cause mortality n_deaths_ = 204, death records	Prospective	1404 (44%)	69 (64–73)	Germany	HR_all-cause mortality_ = 0.72 (0.57, 0.91) for PDI
Shan, 2023 [74]	Total mortality n_deaths_ = 22,900 Cause-specific n_CVDdeaths_ = 6641, death records	Prospective	NHS: 75,230 (100%) HPFS: 44,085 (0%)	NHS: 50.2 ± 7.2 HPFS: 53.3 ± 9.6	US	HR_all-cause mortality_ = 0.86 (0.83, 0.89) comparing extreme quintiles of hPDI HR_CVDmortality_ =0.94 (0.89, 0.99) per 25 percentile increase in hPDI
Wang, 2023 [75]	Total mortality n_deaths_ = 31,136 Cause-specific n_CVDdeaths_ = 9751, death records	Prospective	315,919 (8.1%)	65.5 (na)	US	HR_all-cause mortality_ = 0.75 (0.71, 0.79) for PDI, 0.64 (0.61, 0.68) for hPDI and 1.41 (1.33, 1.49) for uPDI comparing extreme deciles Similar significant associations for CVD mortality

hPDI = healthful plant-based diet index, uPDI = unhealthful plant-based diet index, PDI = plant-based diet index, FFQ = Food Frequency Questionnaire, CVD = Cardiovascular disease, NHS = Nurse’s Health Study, HPFS= Health Professional Follow-up Study, CCHS = Canadian Community Health Survey, UK = United Kingdom, US = United States, na = not available.

**Table 5:** Overview over studies assessing the association between the plant-based diet index, cognitive impairment, and gut microbiome.

Study			Population characteristics			
Authors	Outcome, assessment method	Study design	n (% female)	Age in years	Country	Main findings
Baden, 2020 [76]	Health-related quality of life, Self-reported	Prospective	NHS: 50,290 (100%) NHSII: 51,784 (100%)	NHS: 58 ± 7 NHSII: 39 ± 5	US	Per 10-unit higher hPDI β_PCS_ = 0.13 (0.08, 0.19) β_MCS_ = 0.09 (0.03, 0.15) Per 10-unit higher uPDI β_PCS_ = −0.07 (−0.12, −0.02)β _MCS_ = −0.10 (−0.16, −0.05) Positive association of hPDI with PCS was significant among older females, and with MCS in younger females
Liang, 2022 [77]	Cognitive impairment n_cases_ = 1077, MMSE	Prospective	4792 (49.4%)	80.7 ± 9.6	China	HR_CI_ = 1.32 (1.16, 1.50) for lower PDI, 1.46 (1.29, 1.66) for lower hPDI and 1.21 (1.06, 1.38) for higher uPDI Protective effect of overweight was stronger among those with higher PDI (0.74 [0.57, 0.95]) and higher hPDI (0.73 [0.57, 0.94]) and lower uPDI (0.61 [0.46, 0.80]) compared to lower adherence
Liu, 2022 [78]	Cognitive decline, MMSE, cognitive testing	Prospective	3337 (64.0%)	73.7 ± 5.7	US	β_globalCF_ = 0.0183 ± 0.009, β_perceptualspeed_ = 0.0179 ±0.009 and β_episodicmemory_ = 0.0163 ± 0.012 comparing extreme quintile of hPDI in African American participants
Ma, 2023 [79]	Mood	Cross-sectional	333 (66.1%)	40.6 ± 19.9	UK	β_mood_ = 0.663, p = 0.003 for PDI only in children
Van Soest, 2023 [80]	Cognitive ageing, Cognitive testing battery	Longitudinal	658 (41%)	72.1 ± 5.4	Netherlands	No significant association between PDIs and cognitive ageing Potential interaction with fish consumption: β_globalCF_ = 0.12 (0.03, 0.21) per 10-unit increment of PDI only for individuals with high fish consumption
Wu, 2019 [81]	Cognitive impairment n_cases_ = 2443, MMSE	Prospective	16,948 (59.2%)	73.2 ± 6.2	Singapore	OR_CI_ = 0.82 (0.71, 0.94) for PDI and 0.78 (0.68, 0.90) comparing extreme quartiles
Zhou, 2020 [82]	Healthy ageing n_cases_ = 2834, self-reported	Prospective	14,159 (59.0%)	53.3 ± 6.1	Singapore	OR_healthyageing_ = 1.34 (1.18, 1.53) for PDI and 1.45 (1.27, 1.65) for hPDI OR_CI_ = 1.23 (1.06,1.43) for hPDI
Zhu, 2022 [83]	Cognitive function, MMSE	Prospective	6136 (46.3%)	79.5 ± 9.8	China	OR_CI_ = 0.45 (0.39, 0.52) for PDI, 0.61 (0.54, 0.70) for hPDI and 2.03 (1.79, 2.31) for uPDI comparing extreme quartiles
Hamaya, 2020 [86]	Circulating TMAO levels, Blood measurements	Prospective	620 (0%)	67.7 ± 7.7	US	β_TMAO_ = 0.0015 (0.0007,0.023) for hPDI, −0.013 (−0.021, −0.005) for uPDI
Heianza, 2020 [87]	Circulating TMAO levels and CHD incidence n_cases_ = 380, medical records, blood measurement	Prospective case-control	760 (100%)	58.2 ± 6.5	US	RR_CHD_ = 1.33 (1.06, 1.67) per 1 SD increment TMAO, this association was attenuated by hPDI adherence: RR_CHD_ = 1.48 for low adherence vs. 1.25 for high adherence per 1 SD increment of TMAO
Liu, 2021 [88]	Gut microbiota metabolites CAD risk n_cases_ = 608, medical records, blood measurements	Prospective case-control	NHSII: 374 (100%) HPFS: 842 (0%)	NHSII: 45.7 ± 4.1 HPFS: 63.6 ± 8.7	US	OR_CAD_ = 0.58 (0.38, 0.90) for high enterolactone/low TMAO profile participants with this profile had significantly higher hPDI scores (56.0 (55.1, 56.8) compared to those with low enterolactone/high TMAO profile (54.1 [53.3, 54.8])
Miao, 2022 [89]	Gut microbiome composition, Fecal samples	Prospective	3096 (52.3%)	51.5 ± 12.5	China	Higher short-term hPDI associated with higher Shannon’s diversity index and Pielou’s evenness (β = 0.15 and ≈0.20, respectively), Higher long-term PDI associated with lower abundance of Firmicutes (Q5 vs. Q1 β = −0.15 [−0.26, −0.03]) Four gut microbial features of long-term plant diet associated with HDL-C, LDL-C, TG and CRP (q<0.25)

hPDI = healthful plant-based diet index, uPDI = unhealthful plant-based diet index, PDI = plant-based diet index, NHS = Nurse’s Health Study, HPFS= Health Professional Follow-up Study, UK = United Kingdom, US = United States, na = not available.

7DDR: seven-day dietary record, FFQ = Food Frequency Questionnaire, TMAO = trimethylamine N-oxide, CI = Cognitive impairment, CAD = coronary artery disease, CHD = coronary heart disease, MMSE = Mini Mental State Examination.

### 3.1 Cardiovascular disease

Cardiovascular diseases (CVD) describe disorders pertaining the heart and circulatory systems, and prominent examples include coronary heart disease (CHD) and stroke. The WHO lists CVDs as the leading cause of death, with 32% of all global deaths attributable to CVD [20]. Eleven papers on CVD were reviewed [[21], [22], [23], [24], [25], [26], [27], [28], [29], [30], [31]] (Table 1). Comparison of highest vs. lowest hPDI adherence showed higher adherence was associated with a lowered risk of 8–68% for CVD [[22], [23], [24],^26^,^30^], 17–25% for CHD [28,29], 10% for stroke [21,29]. Meanwhile, adherence to an unhealthful plant-based diet was associated with an increase in risk of 21% for CVD, 32% for CHD [28,30]. In case of differing results, studies still reported associations with single food groups or dose-response associations in the expected direction, indicating at least a partial effect [[25], [26], [27],^31^]. The association to lower CVD risk for those adhering to hPDI appeared to be independent of the genetic risk for CVD, as represented by a polygenic risk score, for CVD [23]. Interestingly however, among those with a high genetic risk score for obesity, the association between hPDI and a lower CVD risk was enhanced [24]. Stratified analysis mostly revealed no significant effect modification by age and sex, with only one study reporting stronger effects in women [26]. While mainly no association was found for an overall plant-based diet and CVD risk, most research points towards a lower risk for CVD and related disorders for those adhering to a healthful plant-based diet, and an increased risk for adherence to an unhealthful plant-based diet.

### 3.2 Type 2 diabetes

Type 2 Diabetes Mellitus (T2D) is characterized by elevated blood glucose levels caused by ineffective use of insulin, which may over time damage blood vessels and neurons. Lifestyle, including diet, plays an important part in the prevention of the disease [32]. In total eleven studies were evaluated [15,[33], [34], [35], [36], [37], [38], [39], [40], [41], [42]] (Table 2). Comparison of lowest vs. highest hPDI adherence revealed a 14–46% lower risk of T2D for those with higher hPDI scores, whereas the overall PDI associated with T2D less strong yet in the same direction. Further, per 10-unit increase in hPDI a 10% higher insulin sensitivity was observed [15,26,[33], [34], [35], [36], [37], [38], [39], [40]]. In contrast, the association between higher uPDI adherence and an increased risk for T2D observed in the original publication of the PDI [15] could not be replicated in further studies [[35], [36], [37],^39^,^40^]. This lack of association may suggest that healthy plant foods more strongly lower the risk of T2D than a higher intake in unhealthy plant foods increases said risk. It has previously been found that long-term changes in uPDI were driven by changes in intake of all three food groups: healthful, unhealthful, and animal foods. An unfavorable decrease in the intake of healthy plant foods could have been compensated by a beneficial decrease in potentially harmful animal foods, therefore resulting in no effect [35]. Two studies observed a stronger inverse association of hPDI or PDI with T2D risk in older adults, suggesting an overall or healthful plant-based diet may be especially beneficial for this age group [15,42]. Furthermore, one study observed a stronger association between T2D and PDI among males [42]. In general, uPDI showed no association with T2D risk, while adherence to both, hPDI, and overall PDI, associated with a decreased risk for T2D.

### 3.3 Metabolic risk factors

In total 19 studies evaluated the association between adherence to a plant-based diet and metabolic risk factors: Two of those focused on hypertension as an outcome [40,43]. Four studies focused on metabolic syndrome as well as its components [[44], [45], [46], [47]]. Seven studies focused on adiposity and related biomarkers [[48], [49], [50], [51], [52], [53], [54], [55]], four on dyslipidemia [[56], [57], [58], [59]] and four on several metabolic risk factors including blood lipids and BMI [33,60,61].

#### 3.3.1 Hypertension

Hypertension is defined as blood pressure of 140/90 mmHg or higher. Hypertension is common, yet if untreated may increase the risk for cardiovascular diseases [62]. Among others, diet can pose a risk factor for hypertension. In two studies, adherence to hPDI was associated with a 17–35% lower risk for hypertension [40,43]. Meanwhile, adherence to an unhealthful plant-based diet was associated with 10–44% higher risk for hypertension (Table 3). One study reported stronger effects in females, potentially explained by an effect of estrogen on vascular function [43].

#### 3.3.2 Adiposity and related biomarkers

Obesity, and in particular the metabolically active visceral adipose tissue, represent a well-known risk factor for many diseases, including cardiometabolic diseases and type 2 diabetes risk [32,63] (Table 3). Higher adherence to hPDI was associated with 26% lower odds for obesity and 5% lower visceral adipose tissue volume per 10-unit increase, as well as 0.68 kg less weight gain per SD increase [33,[51], [52], [53]]. Additionally, higher hPDI has been associated with more beneficial levels of adiposity markers, including lower levels of leptin (−7%), insulin (−10%), HbA1c, hsCRP (−14%) and higher levels of adiponectin (+3%), however the available studies are small, warranting replication in larger cohorts [33,49,53]. In contrast, two large cohort studies, could not find an association between adiposity and hPDI adherence, presumably because intake of healthy plant foods was high in the study population, thereby leading to low variation [44,46,47,54]. Similarly, an overall PDI was associated with a lower weight gain, BMI, waist circumference, fasting glucose, insulin resistance in several studies [33,50,52,55]. Notably, an association was also found with a slightly lower fat-free mass index (−0.16 [−0.21, −0.11] per 10-unit increase in PDI), which may suggest that while an overall plant-based diet may be beneficial for weight loss it could also be adverse for the preservation of fat free mass [50]. Meanwhile, a higher adherence to uPDI generally associated with higher levels of leptin (+4.4%) and insulin (+4.8) [33,49]. Two large studies further reports a 63% increased risk for obesity and for abdominal adiposity (HR = 1.46 comparing highest vs. lowest quintile) and 0.36 kg more weight gain per SD increase in uPDI [27,47,52]. In summary, an unhealthful plant-based diet may have a negative influence on adiposity-related biomarkers, abdominal adiposity and long-term weight gain. Further, these findings may support the notion that a healthful plant-based diet, and an overall plant-based diet may contribute to beneficial levels of markers related to adiposity and potentially to lower adiposity.

#### 3.3.3 Dyslipidemia

Dyslipidemia is characterized by abnormal cholesterol and triglyceride levels and constitutes a risk factor for several diseases, including CVD, CHD, and diabetes [64,65]. Although higher adherence to hPDI and PDI was associated with a 37% or 20–22% respectively lower risk for dyslipidemia [56,59], the majority of evidence could not confirm this [[57], [58], [59]] (Table 3). A potential explanation for the lack of association could be that most respective studies were conducted in South Korea, where intake in plant foods is already high, therefore there may be low variation in the sample. However, several studies report an association between hPDI adherence and individual lipid disorders, such as low HDL or elevated LDL or total cholesterol [33,45,53,58,60,66]. Meanwhile, higher adherence to uPDI consistently associated with a 15–48% increased risk for dyslipidemia or individual lipid disorders [46,47,[56], [57], [58], [59], [60], [61],^66^]. The different origins of the participants and concomitant differences in food culture and selection could potentially explain this finding. Out of the cited studies only one assessed an interaction by age, reporting a stronger association between uPDI and dyslipidemia among older adults (>55 years). Authors suggest that this may be on one hand due to ageing-related changes in lipid metabolism increasing vulnerability to dyslipidemia. On the other hand, older adults were found to have a lower variety of foods, including healthy plant foods, in their diet. The fact that older adults tended to have a long-term adherence to this unhealthful plant-based diet, could have resulted in the quality of the food to have a greater impact on blood lipids. For this group increasing their intake of healthy plant foods may lead to improved blood lipid levels [57]. These findings suggest that adherence to an unhealthful plant-based diet may be associated with an increased risk for dyslipidemia or its components, while a healthful and overall plant-based diet is primarily associated with individual lipid disorders.

#### 3.3.4 Metabolic syndrome

Metabolic syndrome describes a combination of conditions, including central adiposity, elevated blood glucose and blood pressure, low levels of HDL and dyslipidemia and is an established risk factor for cardiovascular disease and mortality [67]. The majority of research report no consistent association between hPDI or overall PDI and metabolic syndrome, potentially because the study population is adapted to a traditionally plant rich diet and may therefore not exhibit a significant metabolic response [33,44,47] (Table 3). Meanwhile, two large cohort studies report a 16–54% increased risk for metabolic syndrome for those with high uPDI scores [46,47]. In addition, one study reported sex-specific differences: in males higher uPDI scores were associated with higher odds for hypertriacylglycerolaemia, while in women, higher odds for hypertriacylglycerolaemia, abdominal obesity and high fasting glucose were observed [47]. None of the evaluated studies assessed an interaction with age. While there seems to be an unclear association for a healthful and overall plant-based diet, these results suggests that an unhealthful plant-based diet is associated with an increased risk for metabolic syndrome. Further, differences in between males and females may be taken into account by future research.

### 3.4 Mortality

Whereas studies have found that a plant-based diet may reduce the risk for certain diseases, it is unclear whether this translates into a reduced mortality risk. In total, 13 studies evaluated how adherence to plant-based diets is associated with mortality [25,27,30,31,[68], [69], [70], [71], [72], [73], [74], [75]] (Table 4). All but two further assessed the association with CVD-mortality [31,73], whereas one study only considered CVD-related mortality [27]. In general, studies adjusted for variables indicative of education and income levels, or the study population was selected to increase homogeneity for these variables.

#### 3.4.1 All-cause mortality

Several studies found a healthier plant-based diet to be associated with a 10–36% lower risk for all-cause mortality [25,30,68,69,72,74,75], while an overall plant-based diet was associated with a 5–28% lower mortality risk [25,68,[71], [72], [73],^75^]. Meanwhile, the few studies that found no association suggested on one hand, that there may be a threshold of healthy plant foods in a diet required to reap their benefits and on the other hand that in populations with overall high intake of plant foods variation may be too low to detect benefits [31,70,71]. Furthermore, the categorization of foods as "healthy" or "less healthy" has been questioned, as for example potatoes and fruit juices, classified as “less healthy,” associated with lower mortality [73]. This fact may also contribute to conflicting findings on uPDI adherence and mortality: studies report either no association [25,31,69,70,73], or a 12–41% increased risk in all-cause mortality [30,68,71,72,75]. Studies point towards a decreased risk for all-cause mortality for adherence to a healthful and overall plant-based diet, whereas an unhealthful plant-based diet may potentially be associated with increased mortality risk.

#### 3.4.2 CVD mortality

Higher adherence to hPDI associated with a 9–36% lower risk for CVD mortality, when comparing extreme quantiles of hPDI [25,68,69,74,75], whereas several studies report no association [27,30,[70], [71], [72]]. However, studies reporting no association tended to be smaller with fewer cases of CVD deaths, therefore suffer from a lack of power. Further possible explanations for the conflicting findings may for example include participants potentially changing their diet after being diagnosed with a CVD or by differences in ethnicity and subsequent CVD risk in populations [71,72]. Similarly, highest vs. lowest uPDI adherence was associated with an up to 55% increased CVD mortality risk in a few studies [68,71,72,75], while this was not confirmed by other, mostly smaller studies [25,27,30,69,70]. Lastly, three studies found an overall PDI to associate with 25% lower CVD mortality risk [25,68,75], whereas the remaining and partially smaller studies found no association [27,[70], [71], [72]]. Overall, evidence suggests adherence to a healthful and overall plant-based diet to be associated with a lower risk for CVD mortality and an increased risk for an unhealthful plant-based diet. Considerable inconsistency between study results is noted, highlighting the need for further research.

### 3.5 Cognitive impairment and well-being

Out of the identified publications, eight focused on cognitive function or mood and well-being [[76], [77], [78], [79], [80], [81], [82], [83]]. Cognitive Impairment (CI) describes loss of memory, difficulties in processing and focusing on a task and has been found to be associated with mortality risk in older adults [84,85]. Adherence to a healthful plant-based diet was associated with lower risk for CI or slower cognitive decline as well as increased mental and physical well-being [[76], [77], [78],^81^,[77], [78], [79], [80], [81], [82], [83]], whereas only two smaller studies found no association with mood [79] or CI [80]. Compared to that, adherence to uPDI associated with a higher risk for CI and decreased well-being [76,77,83]. While there is evidence pointing towards a positive effect of a healthful plant-based diet on CI and the gut microbiome. Sample sizes are in some cases small, and findings are in part contradicting, indicating the importance of conduction larger studies in various populations.

### 3.6 Gut microbiome

Four publications assessed the gut microbiome [[86], [87], [88], [89]]. The gut microbiota generates bioactive compounds that may influence host health in a positive way while certain compounds though, such as trimethylamine N-oxide (TMAO), could also adversely affect health and CVD risk [[90], [91], [92]]. Preliminary evidence showed that higher adherence to a healthful plant-based diet was associated with higher levels of enterolactone, a compound associated with lower CAD risk in females, whereas there was no clear association with TMAO. Additionally, hPDI associated with greater species abundance and diversity in the gut [66,88]. Meanwhile, adherence to an unhealthful plant-based diet showed mostly opposite associations with species compared to a healthful and overall plant-based diet [66,89].

## 4 Discussion

### 4.1 Synthesis of the association of PDI with health outcomes and knowledge gaps

**Fig. 1:**
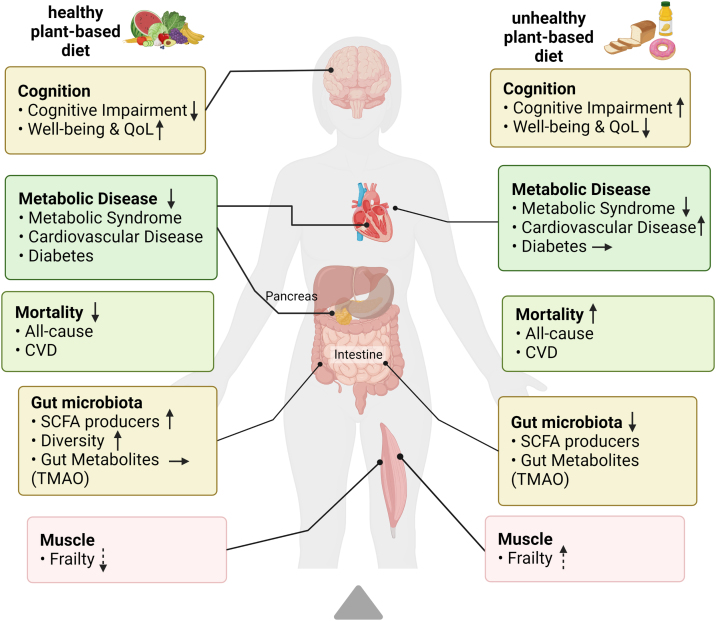
Summary of Evidence on plant-based diet and age-related diseases. Color of text fields indicates amount of available evidence from plenty (green) to little (red), arrows indicate direction of association: upward = positive; downward = negative, dashed arrows indicate lack of evidence. QoL: quality of life, TMAO: trimethylamine N-oxide. Created with BioRender.com.

In summary, amalgamation shows, that adherence to a healthier plant-based diet is associated with a lower risk for metabolic risk factors, diabetes, and cardiovascular disease, while adherence to an unhealthier plant-based diet is associated with a higher risk (Fig. 1). As such, these findings are in agreement with systematic reviews evaluating different types of plant-based diets, including vegetarian and vegan diets, and similar health outcomes [5,93,94]. Effects and associations of the healthful plant-based diet are hypothesized to be mainly due to anti-inflammatory and antioxidant effects of healthful plant foods, as well as their richness in micronutrients and fiber and lower glycemic index [95,96]. Their beneficial effect on health markers may be further explained by an impact on the gut microbiome [49,51]. Fewer studies investigated cognitive impairment and the gut microbiome, indicating that a healthy plant-based diet is associated with lower cognitive impairment, better well-being and greater gut microbiome diversity, and associations with an unhealthful plant-based diet maybe opposite [66,[76], [77], [78], [79], [80], [81], [82], [83],[86], [87], [88], [89]]. We conclude, that the PDI has been applied in various cardiometabolic health studies and has shown consistent associations.

Only few studies examined effects in older adults (>70 years) specifically, for example by means of stratification or interaction analysis, which impedes drawing conclusions on the effect of plant-based diets on health in this age group. Virtually no evidence is available on physical fitness and muscle health. Only one study so far assessed the association between the PDI and frailty [97]. Cancer as an outcome has not been included in this review, as the extent to which it associated with nutrition varies by type of cancer. Moreover, so far only two studies assessed an interaction of a healthful plant-based diet and genetic risk, which may be further explored in future studies to identify populations who may benefit in particular from following a healthful plant-based diet. Studies focusing on the gut microbiome or cognitive impairment as an outcome are quite small or are often conducted in Chinese populations, making it difficult to draw conclusions for populations following a Western diet. PDI is suitable as a tool to gain a better understanding of how different types of plant-based diet affect health and how this may reflect in the gut microbiome. We see the need for future studies applying the PDI in different populations around the world, patient groups, older adults and genetic risk groups, as potentially vulnerable segments of the population for which energy deficits may be at stake.

### 4.2 Benefits of applying the PDI

Our review and consequent conclusions emphasize the benefits of applying a diet score, such as the PDI, across multiple studies, as it allows for the comparison of results.

The fact that the PDI is a tool, based on items found on dietary assessment methods make it highly flexible, so it can be used in diverse populations. Rather than for example a Mediterranean diet score, it does not rely on specific foods, such as olive oil, which are not commonly consumed in many parts of the world. It can therefore contribute to obtain an understanding on what a healthful plant-based diet can look like based on foods locally available and consumed in a population.

Additionally, by differentiating between healthy and unhealthy plant foods it allows researchers to gain a better understanding on the role of diet quality as well as the proportion of animal to plant foods. Variations of the index have been applied in the past to investigate nuances of plant-based diet adherence, for example, by scoring presumably healthy animal foods positively. Generally, these adaptations to the index were not associated with strong changes in association with disease risk, suggesting high intake of high-quality plant foods may be more important in lowering disease risk [15,98]. Conducting sensitivity analysis, for example excluding food groups one at a time, allows to gage the extent to which a single food group may explain observed associations. At the same time, the effects of a gradual reduction in animal food intake can be taken into account, allowing for a nuanced approach, revealing that a complete exclusion of animal foods may not be necessary for beneficial effects on health.

Further, studies comparing different healthy dietary patterns have found the hPDI to be associated with diabetes incidence independent of other healthy, mostly unprocessed dietary patterns, suggesting it captures a unique aspect of plant-based diets, not covered by other diet patterns [15]. It is so far unclear where this uniqueness lies. More research is therefore needed to understand the unique properties of the plant-based diet index.

In summary, we conclude that despite its weaknesses, for generating knowledge on plant-based diet patterns moving forward, the PDI is a useful tool.

### 4.3 Recommendations for future research applying the PDI

There are several points to be considered, when applying the plant-based diet index in epidemiological studies. The calculation of the index can vary between studies, depending on the underlying dietary assessment method used, reflecting regional or cultural differences in diet and national dietary guidelines. Missing information on food groups may explain lack of association in previous studies [57]. When starting novel data collection, it is therefore advised to ensure information on all major food groups is collected. Secondly, the index uses a quantile-based approach rather than relying on absolute intake values for scoring, as common with other dietary scores. While this allows for ranking participants within one study based on their adherence to the index, it may hamper comparison between studies, as the absolute intakes of foods may differ between people with the same PDI score. Further difficulties may include the appropriate categorization of compound items, as well as different methods of energy adjustment, which may introduce different forms of bias. Therefore, when working with the PDI the underlying calculation method should be considered when interpreting results.

According to the opposite scoring pattern of the healthful and unhealthful plant-based diet index, it stands to reason that also the associations of both indices with health outcomes should be opposite. However, this is not always the case. Several mechanisms could explain this phenomenon. First, in populations with an overall high intake of healthy plant-foods low variation may lead to a lack of association. However, this does not necessarily imply that a high intake in unhealthy plant-foods is unproblematic. Secondly, differences in how studies construct the index may play a role. The dietary data used to construct the index may not allow for a clear separation of unhealthy and healthy plant foods thereby lowering discriminatory power between both indices [46]. Lastly, the scoring of the PDI may partly be based on faulty assumptions: one study reported potatoes, typically considered an unhealthy plant food, to be associated with lower incident CVD. By reverse scoring this item, a potential association of the hPDI with CVD incidence could have been attenuated. This leads the authors to conclude that the association between certain foods and disease risk may not yet be fully understood and that future study may consider a different categorization [25].

Suggestions to improve the use of the PDI in future studies include reporting changes made to the originally published index based on local food culture in the method section and providing reasons behind these changes [99]. Ideally, authors can further give an estimation of the effect of changes, such as additions of food groups have on the index and its associations with health outcomes, e.g., via sensitivity analysis of single food groups [43,83]. Thus, while changes to the index may make it a more valid tool for a specific population, this also impedes comparability to previous results. Detailed description of the PDI calculations and deviation of the original version are recommended.

A further recommendation lies in reporting absolute intakes in food groups, e.g., by quantile of PDI. In previous studies, it has been suggested that a minimum amount of plant foods might be necessary to observe a meaningful effect on health [70]. Such a threshold, if it exists, could explain why no effects have been reported in populations with low plant-based diet adherence [31]. Knowledge of absolute intakes of foods, or potentially also nutrients per quantile of PDI, could give an indication of where this threshold lies and may further become relevant when studying vulnerable populations, such as older adults, as nutritional needs may change over the life course: Including information about absolute intakes may therefore contribute to an increased understanding of the effect of plant-based diets in different populations.

As the index is mainly applied in cohort studies, it is recommended to account for changes in food regulations and food compositions. A prominent example for this is margarine: it was previously a source of unfavorable trans fats due to its manufacturing process, whereas nowadays changes in legislation or societal pressure have led to a reduction of trans fats in foods in many European countries [99,100].

Lastly, only few studies have considered changes in plant-based diet adherence [35,49,52,54,68,76]. Especially regarding the gut microbiome, existing literature suggests, that duration of adherence may impact species diversity and related levels of health markers differently [89]. Investigating the role of long-term diet adherence and changes in diet intake may yield insights into required adherence before effects on health can be observed.

## 5 Conclusion

In summary, the plant-based diet index has been used in numerous studies, mostly focused on metabolic health outcomes. Generally, a healthier plant-based diet is associated with a lower risk for metabolic diseases, underlining its contribution to promote healthy ageing. Association of the plant-based diet index with cognitive impairment, well-being, the gut microbiome, physical function, and loss of muscle mass, influences of genetic predisposition and effects in vulnerable populations are less well studied, highlighting key areas for future research. Further, relevant outcomes for future research applying the plant-based diet index include inflammatory biomarkers. For future research our recommendations include (1) providing a transparent method description including an explanation for diverting from the original scoring pattern, (2) give an estimation of how changes made to the index may impact results, (3) provide an overview over absolute intakes (4) adjust categorization of food items to current literature and dietary habits of the study population, (5) assess associations in different age groups for an increased understanding of effects also among older adults. If these aspects are considered, we rate the plant-based diet index as a useful tool for quantifying plant-based diet adherence and summarizing findings on plant-based diets and health.

## Conflict of interests

The authors declare that they have no known competing financial interests or personal relationships that could have appeared to influence the work reported in this paper.

## Funding

K.A. Schorr and V. Agayn: This work has received funding from the European Union’s Horizon 2020research and innovation framework under the Marie Sklodowska-Curie grant agreement No. 860173. P.E. Slagboom, M. Beekman, and L.C.P.G.M de Groot: received funding from the VOILA Consortium (ZonMw 457001001).
