# Supplementary material for: A plant-based diet index to study the relation between diet and disease risk among adults: a narrative review
Source: J Nutr Health Aging. 2024 May 29;28(6):100272. doi: 10.1016/j.jnha.2024.100272 (PMC12275787; doi:10.1016/j.jnha.2024.100272)
Supplement: Supplementary file 1 [file mmc1.docx]

Supplement A plant-based diet index to study the relation between diet and disease risk among adults: a narrative review

**Table S1 Food groups, example items and scoring of PDI, based on Satija et al (2017)**

|  |  | PDI | hPDI | uPDI |
| --- | --- | --- | --- | --- |
| healthful |  |  |  |  |
| Vegetables | Fresh vegetables, vegetable juice, sweet potatoes, garlic | **+** | **+** | **-** |
| Fruits | fresh fruit, raisins | **+** | **+** | **-** |
| Nuts | Nuts, peanut butter |  |  |  |
| Legumes | Beans, Soy, Tofu, lentils | **+** | **+** | **-** |
| Wholegrains | Whole grain cereal, oatmeal, brown rice, whole wheat pasta | **+** | **+** | **-** |
| Vegetable Oils | Vegetable oils used for cooking, oil-based salad dressings | **+** | **+** | **-** |
| Tea & Coffee | Incl. decaffeinated coffee | **+** | **+** | **-** |
| Less healthful |  |  |  |  |
| Refined grains | White bread, refined cereal, white rice, pasta | **+** | **-** | **+** |
| Fruit Juices | Apple cider, fruit juices | **+** | **-** | **+** |
| Potatoes | French fries, baked potatoes, chips | **+** | **-** | **+** |
| Sweets & Desserts | Chocolate, cookies, pie, jams or jellies | **+** | **-** | **+** |
| Sugar sweetened beverages | Colas and carbonated beverages with sugar | **+** | **-** | **+** |
| Animal food groups |  |  |  |  |
| Animal fats | Butter, lard | **-** | **-** | **-** |
| Eggs | Eggs | **-** | **-** | **-** |
| Dairy | Milk, cheese, ice cream, yogurt | **-** | **-** | **-** |
| Fish | Dark meat fish, other fish, seafood | **-** | **-** | **-** |
| Meat & Meatproducts | Chicken, pork, beef, bacon, sausages | **-** | **-** | **-** |
| Miscellaneous animal-based foods | Pizza, mayonnaise | **-** | **-** | **-** |

Material S1: Search strategy

(plant-based dietary pattern [all fields] OR plant-based diet index [all fields] OR plant-based diet indices [all fields]) AND ("Gastrointestinal Microbiome"[Mesh] OR gut microbiome[Title/Abstract] OR "Cardiovascular Diseases"[Mesh] OR cardiovascular disease[Title/Abstract] OR "Cognition"[Mesh] OR cognitive impairment[Title/Abstract] OR physical function[Title/Abstract] OR "Frailty"[Mesh] OR "Quality of Life"[Mesh] OR well-being[Title/Abstract] OR "Aging"[Mesh] OR "Aged"[Mesh] OR elderly[Title/Abstract])

Figure S1 Flowchart of study inclusion

730 studies through search in PubMed from 2016-2023

61 studies identified after title and abstract screening

11 studies identified through other sources

11 Excluded for not fitting inclusion criteria

50 studies identified

61 studies included in review

Table S2 Overview over studies assessing the association between the plant-based diet index and cardiovascular diseases

| Study | | | Population Characteristics | | | PDI | | | |  |
| --- | --- | --- | --- | --- | --- | --- | --- | --- | --- | --- |
| Author, year [ID] | Outcome, assessment method | Study design | N (% female) | Age in years | Country | Version | Dietary assessment | Mean±SD,  Median [range],  Median quantile 1-x | Energy-adjustment | Main Findings |
| Baden,2021 [21] | Stroke  n_total_=6241  n_ischemic_=3015  n_hemorrhagic_=853,  medical records | Prospective | NHS: 73,890 (100%)  NHSII: 92,352 (100%)  HPFS: 43,266 (0%) | NHS: 51±7  NHS II: 37±5  HPFS:54±10 | US | - Original hPDI, uPDI and PDI - 18 food groups - range 18-90 | FFQ | PDI: 57.2±9.3  hPDI: 59.2±7  uPDI:  55.2±8.5 | Covariate | HR_total_=0.90 [0.83-0.98] comparing extreme quintiles of PDI |
| Chen, 2022 [22] | Cardiovascular disease  n_CVDevents_=232,  self-reported | Prospective | 10,293 (57.9%) | 40.7±0.4 | US | - Revised hPDI - 15 food groups - Range 15-75 | 24h diet recall | Q1-3hPDI:  39-52 | Covariate | RR_CVD_=0.74 (0.60-0.93) per 1-SD increment of hPDI |
| Heianza, 2020 [23] | Cardiovascular disease  n_CVDevents_=1812,  medical/death registry | Prospective | 156,148 (54.5%) | 56±8 | UK | - Revised hPDI - 17 food groups - Range 17-85 | 24h diet recall | 56.6±1.8 | Covariate | HR_CVD_=0.90 (0.84, 0.97) per 10-unit increment of hPDI, no interaction with GRS |
| Heianza, 2021 [24] | Cardiovascular disease  n_CVDevents_=1033,  medical/death registry | Prospective | 121,799 (57.4%) | 55.1±7.9 | UK | - Revised hPDI - 17 food groups - Range 17-85 | 24h diet recall | 56±2 | Covariate | HR_MI_=0.54 (0.39, 0.74) among high GRS group for hPDI, p_interaction GRSXhPDI_<0.001 on BMI |
| Kim, 2019 [25] | Cardiovascular disease  n_CVDevents_=4381,  self-reported, hospital records | Prospective | 12,168 (55.9%) | 53.8±5.7 | US | - Revised PDI. hPDI, uPDI - 17 food groups - Range 17-85 | FFQ | Q1-5  PDI 47-66  hPDI 29-61  uPDI 43-59 | Residual method,  Covariate | HR_CVD_=0.84 (0.75,0.92) for highest vs lowest quintile of PDI |
| Kouvari, 2022 [26] | Cardiovascular disease risk  n_CVDevents_=317,  self-reported, death registries | Prospective | 2020 (45.8%) | 39.8±10.9 | Greece | - Revised PDI. hPDI, uPDI - 20 food groups - Range 20-100 | FFQ | Q1-3  PDI 59-68  hPDI 49-51  uPDI 48-57 | Covariate | HR_CVD_=0.32 [0.16, 0.63] for hPDI comparing extreme tertiles |
| Lazarova, 2022 [27] | Cardiovascular disease (CCHS 2004)  n_events_=748,  hospital and death records | Cross-sectional | CCHS 2004: 6771 (n.a.) | n.a. | Canada | - Revised hPDI, uPDI and PDI - 18 food groups - range 18-90 | 24h diet recall | PDI 39.11± 0.14  hPDI: 43.2± 0.16  uPDI: 41.8± 0.16 | Covariate | No significant association with CVD risk |
| Satija, 2017 [28] | Cardiovascular disease  n_CHD_=8631,  medical and death records | Prospective | NHS: 73,710 (100%)  NHSII: 92,329 (100%)  HPFS: 43,259 (0%) | NHS: 50.0±7.1  NHSII: 36.3±4.6  HPFS: 53.3±9.5 | US | - Original hPDI, uPDI and PDI - 18 food groups - range 18-90 | FFQ | Q1-10  PDI: 44-66  hPDI: 42-67  uPDI: 42-68 | Covariate | HR_CHD_=0.92 (0.83,1.01), 0.75 (0.68, 0.83) and 1.32 (1.20,1.46) comparing extreme deciles of PDI, hPDI and uPDI, respectively |
| Shan, 2020 [29] | Cardiovascular disease  n_CHD_=18,092  n_stroke_=5687,  medial and death records | Prospective | NHS: 74,930 (100%)  NHSII: 90,864 (100%)  HPFS: 43,339 (0%) | NHS: 50.2±7.2  NHSII 36.1±4.7  HPFS 53.2±9.6 | US | - Original hPDI - 18 food groups - range 18-90 | FFQ | Q1-5  44.6-65.4 | Covariate | HR_CVD_=0.86 (0.82,0.89),  HR_Stroke_=0.92 (0.85,1.00),  HR_CHD_=0.84 (0.80,0.87) per 25-percentile higher hPDI |
| Thompson, 2023 [30] | Cardiovascular disease  n_CVD_=6890  medical and death records | Prospective | 126,394 (55.9%) | 56.1±7.8 | UK | - Revised hPDI and uPDI - 17 food groups | 24h diet recall | Q1-4  hPDI: 47.7-63.4  uPDI: 46.6-61.8 | Covariate | HR_CVD_=0.92 (0.86, 0.99), comparing extreme quartiles of hPDI |
| Weston, 2022 [31] | Cardiovascular diseasen_CVDevents_=293,  Self-reported, hospital records | Prospective | 3635 (64.3%) | 53.8±12.5 | US | - Revised PDI, hPDI, uPDI - 18 food groups - range 18-90 | FFQ | Q1-3  PDI: 48-61  hPDI: 48-60  uPDI: 48-61 | Residual method | No significant associations |

hPDI=healthful plant-based diet index, uPDI=unhealthful plant-based diet index, PDI=plant-based diet index, FFQ=Food Frequency Questionnaire, T2D=Type 2 Diabetes, WC=Waist Circumference, FBS=Fasting blood sugar, GRS=Genetic Risk Score, HPFS= Health Professional Follow-up Study, CCHS= Canadian Community Health Survey, UK= United Kingdom, FI= Finland, NZ= New Zealand, BG= Bulgaria, AU= Australia, US= United States, na= not available

Table S3 Overview over studies assessing the association between the plant-based diet index and Type 2 Diabetes

| Study | | | | Population characteristics | | | PDI | | | |  |
| --- | --- | --- | --- | --- | --- | --- | --- | --- | --- | --- | --- |
| 1st author | Outcome, assessment method | Study design | N (% female) | | Age in years | Country | Version | Dietary assessment | Mean±SD,  Median [range],  Median quantile 1-x | Energy-adjustment | Main Findings |
| Bhupathiraju, 2022 [33] | Cardiometabolic risk factors,  Blood draw, anthropo-metry | Cross-sectional  Prospective | 891 (47.2%)  735 (na) | | 55.2±0.64 | South Asia | - Revised PDI, hPDI, uPDI - 20 food groups - Range 20-100 | FFQ | 61,15 ± 0.18 [SEM] | Covariate | Per 5-uni increase in PDI:  β_fasting glucose_=1.03±0.35,  β_HOMA-IR_= -3.46±1.65,  Per 5-unit increase in hPDI:  β_HbA1c_=-0.43±0.14,  β_HOMA-IR_= -4.02±1.42,  OR_T2D_=0.82 (0.67,1.00) |
| Chen, 2018 [34] | Type 2 Diabetes  n_cases_=5207,  self-reported | Prospective | 45,411 (55%) | | 55.0 [45-74] | Singapore | - Revised PDI, hPDI - 15 food groups - Range 15-75 | FFQ | PDI: 39.9 ± na  hPDI: 45.3 ± na | Residual method | HR_T2D_=0.83 (0.76,0.92) for PDI and 0.81 (0.75, 0.89) for hPDI for highest vs lowest quintile |
| Chen, 2021 [35] | Type 2 Diabetes  n_cases_=12,627,  question-naire based on official criteria | Prospective | NHS: 76,530 (100%)  NHSII: 81,569 (100%)  HPFS: 34,468 (0%) | | NHS: 58.1±7.9  NHSII: 41.1±5.4  HPFS: 57.5±9.7 | US | - PDI, hPDI - 18 food groups - Range 18-90 | FFQ | PDI: 55 ± 6,  hPDI: 55 ± 7 | Covariate | HR_T2D_=1.12 (1.05,1.20) for PDI and 1.23 (1.16, 1.31) for hPDI for largest decrease (>10%) vs stable indices |
| Chen, 2018 [36] | Type 2 Diabetes  n_cases_=642,  blood measure-ments | Prospective | 6798 (58.7%) | | 62.7±7.8 | Nether-lands | - Revised PDI - 23 food groups - Range 0-92 | FFQ | 49.3 ± 7.1 | Covariate | HR_T2D_=0.87 (0.79,0.99), β_insulin resistance_= -0.05 (-0.06, -0.04) for PDI per 10-unit increase |
| Flores, 2021 [37] | Type 2 Diabetes  n_cases_=134,  blood measure-ment | Prospective | 646 (72%) | | 55.5±0.5 | Puerto Rico | - Original PDI, hPDI, uPDI - 18 food groups - Range 18-90 | FFQ | Q1-3PDI: 48-61  hPDI: 46-61  uPDI: 48-62 | Covariate | HR_T2D_=0.54 (0.31,0.94) for hPDI for comparing highest vs lowest tertile |
| Goode, 2023 [38] | Insuline Sensitivity,  Blood measure-ments | Prospective | 667 (50.2%) | | 31.5±2.6 | Australia | - Revised hPDI - 17 food groups - Range 17-85 | FFQ | hPDI 51.0±7 | Covariate, residual method | β_insulin-sensitivity_ = 0.11 (0.05, 0.17) between-person and 0.10 (0.04, 0.16) within-person effect for hPDI |
| Kim, 2022 [39] | Type 2 Diabetes  n_cases_=977,  blood measure-ments | Prospective | 7363 (55%) | | 52±8.5 | South Korea | - Revised PDI, hPDI, uPDI - 17 food groups - Range 17-85 | FFQ | Q1-4  PDI: 44-58  hPDI: 43-59  uPDI: 43-62 | Covariate, residual method, adjustment of food groups | HR_T2D_=0.86 (0.77,0.95) for hPDI for comparing highest vs lowest tertile |
| Laouali, 2021 [40] | Type 2 Diabetes  n_T2D_=3292),  self-reported | Prospective | 74,552 (100%) | | 52.9+6.7 | France | - Original hPDI, uPDI and PDI - 18 food groups - Range 18-90 | FFQ | PDI 51.7±6.12 | Covariate | HR_T2D_=0.71 (0.63,0.79) for PDI and 0.74 (0.67, 0.83) for hPDI |
| Satija, 2016 [15] | Type 2 Diabetes  n_cases_=16,162 | Prospective | NHS: 69,949 (100%)  NHSII: 90,239 (100%)  HPFS: 40,539 (0%) | | NHS: 50±7  NHSII: 36±5  HPFS: 53±9 | US | - Original hPDI, uPDI and PDI - 18 food groups - Range 18-90 | FFQ | Q1-10PDI: 44-66  hPDI: 42-67  uPDI: 42-68 | Covariate | HR_T2D_=0.80 (0.74,0.87), 0.66 (0.61, 0.72), 1.16 (1.08,1.25) for PDI, hPDI and uPDI, respectively for comparing extreme deciles |
| Yang, 2021 [41] | Type 2 Diabetes  n_cases_=na,  self-reported | Prospective | 37,985 (60.7%) | | 55.7±12.2 | China | - Revised PDI - 12 food groups - Range 12-48 | FFQ | NA | Covariate | OR_T2D_=0.88 (0.79, 0.98) for PDI comparing extreme quartiles |
| Zhang, 2023 [42] | Type 2 Diabetes  n_cases_=7654,  blood measure-ments | Cross-sectional | 50,694 (59.6%) | | 55.3±9.7 | China | - Revised PDI - 10 food groups - Range 12-60 | FFQ | 46.0 ± 3.85 | - | OR_T2D_=0.83 (0.75,0.92) for high CVD risk population and 0.80 (0.74,0.87) for non-high CVD risk population comparing extreme quartiles |

hPDI=healthful plant-based diet index, uPDI=unhealthful plant-based diet index, PDI=plant-based diet index, FFQ=Food Frequency Questionnaire, T2D=Type 2 Diabetes, WC=Waist Circumference, FBS=Fasting blood sugar, GRS=Genetic Risk Score, HPFS= Health Professional Follow-up Study, CCHS= Canadian Community Health Survey, UK= United Kingdom, FI= Finland, NZ= New Zealand, BG= Bulgaria, AU= Australia, US= United States, na= not available

Table S4: Overview over studies assessing the association between the plant-based diet index and metabolic risk factors

| Study | | | Population Characteristics | | | PDI | | | |  |
| --- | --- | --- | --- | --- | --- | --- | --- | --- | --- | --- |
| 1st author | Outcome,  Assessment method | Study design | N (% female) | Age in years | Country | Version | Dietary assessment | Mean±SD,  Median [range],  Median quantile 1-x | Energy-adjustment | Main Findings |
| Kim, 2021 [43] | Hypertension  n_events_=2244,  measured, self-reported or diagnosed | Prospective | 5639 (53.3%) | 50.6±8.5 | South Korea | - Revised PDI. hPDI, uPDI - 17 food groups - Range 17-85 | FFQ | PDI: 51 (31-70)  hPDI: 51 (28-72)  uPDI: 53 (29-75) | Adjustment of food groups,  Covariate | HR_Hypertension_=0.65 (0.57, 0.75) for hPDI and 1.44 (1.24, 1.67) for uPDI comparing extreme quintile |
| Laouali, 2021 [40] | Hypertension  n_Hypertension_=12,504,  self-reported | Prospective | 74,552 (100%) | 52.9+6.7 | France | - Original hPDI, uPDI and PDI - 18 food groups - Range 18-90 | FFQ | PDI 51.7±6.12 | Covariate | HR_Hypertension_=0.89 (0.44,0.94) for PDI, 0.83 (0.78, 0.88) for hPDI and 1.10 (1.04, 1.17) for uPDI comparing extreme quintiles |
| Lazarova, 2022 [27] | Obesity | Cross-sectional | CCHS 2004: 6771 (n.a.) | n.a. | Canada | - Revised hPDI, uPDI and PDI - 18 food groups - Range 18-90 | 24h diet recall | PDI 37.3 ± 0.34  hPDI: 44.3± 0.15  uPDI: 40.7± 0.15 | Covariate | OR_obesity_=1.63 (1.30-2.05) for unhealthiest vs healthiest quartile |
| Bhupathiraju, 2022 [33] | Cardiometabolic risk factors,  Blood draw, anthropometry | Cross-sectional  Prospective | 891 (47.2%)  735 (na) | 55.2±0.64 | South Asia | - Revised PDI, hPDI, uPDI - 20 food groups - Range 20-100 | FFQ | 61,15 ± 0.18 [SEM] | Covariate | Per 5-unit increase in PDI:  OR_Obesity_=0.86 (0.77,0.97)  β_LDL-C_= -0.08±0.02  Per 5-unit increase in hPDI:  β_visceral fat_= -2.55±0.92,  β_adiponectin_= 2.32±1.08  OR_Obesity_=0.88 (0.80,0.97)  β_LDL-C_= -0.04±0.02  β_Adiponectin_= 2.32±1.08  Per 5-unit increase in uPDI:  β_LDL-C_= -0.04±0.02 |
| Amini, 2021 [44] | Metabolic Syndrome  n_cases_=95,  anthropo-metry, blood measurements | Cross-sectional | 178 (71%) | 67.0±6.1 | Iran | - Original hPDI, uPDI and PDI - 18 food groups - Range 18-90 | FFQ | PDI: 53.8±3.0  hPDI: 53.7±2.5  uPDI: 54.2±3.1 | Covariate | No significant association |
| Jafari 2023 [45] | Metabolic Syndrome  n_cases_=607,  anthropo-metry, blood measurements | Cross-sectional | 2225 (46.7%) | 45.6±8.2 | Iran | - Revised hPDI, uPDI and PDI - 18 food groups - Range 18-180 | FFQ | NA | Covariate | OR_metS_=0.67 (0.52, 0.86) for highest vs lowest tertile of hPDI |
| Kim, 2020 [46] | Metabolic Syndrome  n_cases_=2583,  NCEP-ATP III classification, anthropometry, blood measurements | Prospective | 5646 (48.3%) | 51.0±8.6 | South Korea | - Revised hPDI, uPDI and PDI - 17 modified food groups - Range 17-85 | FFQ | Q1-5  PDI: 45-58  hPDI: 44-59  uPDI: 43-60 | Residual method, Covariate | OR_obesity_=1.23 (1.06-1.42) for extreme quintiles of uPDI |
| Kim, 2021 [47] | Metabolic Syndrome  n_cases_=3367,  anthropometry, blood measurements | Prospective | 14,450 (61.3%) | 41.3±0.4 | South Korea | - Original PDI, hPDI and uPDI - 18 modified food groups - Range 18-90 | FFQ | Q1-5  PDI: 45-60  hPDI: 46-65  uPDI: 46-67 | Adjusted food groups and Covariate | HR_obesityS_=1.46 (1.25-1.71) for extreme quintiles of uPDI |
| Asoudeh, 2023 [48] | Adiposity,  Anthropo-metry | Cross-sectional | 6724 (57%) | 36.8±8.08 | Iran | - Original PDI - 18 food groups - Range 18-90 | FFQ | NA | Covariate | No significant associations |
| Baden, 2019 [49] | Adiposity-related biomarkers,  Blood measure-ments | Prospective | 831 (100%) | 45±5 | US | - Original PDI, hPDI and uPDI - 18 food groups - Range 18-90 | FFQ | PDI: 55.9 ± 4.8  hPDI: 55.2 ± 5.7  uPDI: 54.0 ± 5.7 | Covariate | Per 10-point higher hPDI:  Cross-sectional  Leptin: -7.2% (-11.0,-3,1),  Insulin: -10.0% (-14.2, -5.6)  hsCRP: -13.6% (-20.5, -6.1)  sOB-R: 1.9% (0.3,3.7)  Adiponectin: 3.0% (0.4, 5.7)  Longitudinal:  Leptin: -7.7% (-13.6, -0.4)  hsCRP: -17.8% (-26.3, -8.4)  Per 10-point higher uPDI: |
| Chen, 2019 [50] | Adiposity,  anthropometry | Prospective | 9633 (58%) | 64.2±8.7 | Nether-lands | - Revised PDI - 23 food groups - Range 0-92 | FFQ | Median [IQR]  49.0 [44.0–53.0] | Covariate | Per 10-unit higher PDI:  β_BMI_=-0.70 kg/m^2^ (-0.81,-0.59)  β_WC_= -2.0 cm (-2.3, -1.7)  β_FMI_= -0.66 kg/m^2^ (-0.80, -0.52)  β_BF%_= -1.1 points (-1.3, -0.84) |
| Ratjen, 2020 [51] | Adipose tissue volume,  MRI | Cross-sectional | 578 (43%) | 62 [55-71] | Germany | - Original PDI, hPDI and uPDI - 18 food groups - Range 18-90 | FFQ | Median [IQR]  PDI: 54 [50–58]  hPDI: 54 [49–59]  uPDI: 54 [49–59] | Covariate | Per 10-unit higher hPDI  -4.9% (-8.6, -2.0) visceral adipose tissue |
| Satija, 2019 [52] | Weight Change,  Self-reported | Prospective | NHS: 46,790 (100%)  NHSII: 59,217 (100%)  HPFS: 20,975 (0%) | NHS: 52±7.1  NHSII: 37±4.4  HPFS: 50±7.7 | US | - Original PDI, hPDI and uPDI - 18 food groups - Range 18-90 | FFQ | PDI: 55 ± 6.4  hPDI: 55 ± 7.4  uPDI: 55 ± 7.3 | NA | Per 1-SD increase in PDI  0.04kg (0.05, 0.02) and in hPDI 0.68kg (0.69, 0.66) less weight gain and 0.36 (0.34, 0.37) more weight gain for uPDI |
| Shahavandi, 2020 [53] | Adiposity,  anthropometry | Cross-sectional | 270 (56.3%) | 36.5±13 | Iran | - Original PDI, hPDI and uPDI - 18 food groups - Range 18-90 | FFQ | NA | Covariate | OR_visceralAdiposity_=5.7 (1.15, 28.10) for extreme deciles of uPDI |
| Waterplas, 2020 [54] | BMI, WC, blood lipids, anthropometry, blood measurements | Prospective | 650 (51.1%) | 46±9.2 | Belgium | - Original PDI, hPDI and uPDI - 18 food groups - Range 18-90 | FFQ | PDI: 53.7 ±5.7  hPDI: 54.6 ± 6.0  uPDI: 54.1± 6.5 | Residual method | β_BMI_=0.135 for increases in PDI |
| Zhu, 2021 [55] | Weight maintenance,  cardiometabolic risk factors,  DXA, blood measurements, anthropometry | Prospective | 710 (69.2%) | 57 (46-63) | FI, UK, BG, NZ, AU | - Revised PDI - 11 food groups | Food records | NA | Covariate | Δbodyweight -0.25 (-0.48, -0.002) for PDI adherence |
| Lee, 2021 [56] | Dyslipidemia  n_cases_=2995,  blood measurements | Prospective | 4507 (58.7%) | 51.8±8.9 | South Korea | - Revised PDI, hPDI, uPDI - 17 food groups - Range 17-85 | FFQ | Q1-5:  PDI: 44-54  hPDI: 43-59  uPDI:  44-62 | Covariate, adjustment of food groups | HR_dyslipidemia_=0.78 (0.69, 0.88) for PDI, 0.63 (0.56, 0.70) for hPDI and  1.48 (1.30,1.69) for uPDI when comparing extreme quintiles |
| Song, 2021 [57] | Dyslipidemia  n_cases_=48,166,  blood measurements | Prospective | 147,945 (62.9%) | 53.2±8.2 | South Korea | - Revised PDI, hPDI, uPDI - 17 food groups - Range 17-85 | FFQ | NA | Covariate, adjustment of food groups | HR_dyslipidemia_=1.15 (1.11,1.20) for extreme quintiles of uPDI |
| Shin, 2021 [58] | Dyslipidemia  n_cases_=6658 | Cross-sectional | 14,167 (61.8%) | 40.8±0.1 | South Korea | - Revised PDI, hPDI, uPDI - 17 food groups - Range 17-85 | FFQ | NA | Covariate, adjustment of food groups | OR_dyslipidemia_=1.22 (1.05, 1.41),  OR_highTG_=1.48 (1.21, 1.81),  OR_lowHDL_=1.16 (1.00,1.35) for extreme quintiles of uPDI |
| Wang, 2023 [59] | Dyslipidemia  n_cases_=1501,  blood measurements | Cross-sectional | 4096 (55.1%) | 51.23±10.2 | China | - Revised PDI, hPDI and uPDI - 18 food groups - Range 18-180 | FFQ | NA | NA | OR_dyslipidemia_=0.80 (0.66–0.97) for PDI comparing quintile 4 vs quintile 1  OR_lowHDL_= 0.64 (0.49–0.82) for PDI, 0.66 (0.50–0.87) for hPDI, 1.35 (1.04, 1.74) for uPDI |
| Lotfi, 2022 [60] | Cardiometabolic risk factors,  Anthropometry, blood measurements | Cross-sectional | 3678 (na) | 55.6±7.9 | Iran | - PDI, hPDI and uPDI - 18 food groups, revised scoring - Range 18-90 | FFQ | NA | Covariate | OR_FBS_=0.42 (0.33, 0.53) for PDI,  OR_totalChol_=0.80 (0.65, 0.98) for hPDI,  OR_FBS_=1.23 (1.00, 1.53) and  OR_totalChol_=1.23 (1.01,1.49),  OR_FBS_=1.39 (1.13, 1.71) for uPDI, |
| Shirzadi, 2022 [61] | Cardiovascular risk factors,  Anthropometry, blood measurements | Cross-sectional | 371 (100%) | 30.7±6.9 | Iran | - Revised PDI, hPDI and uPDI - 19 food groups | FFQ | NA | Covariate | Lower LDL-C in Tertile 3 vs Tertile 1 of PDI  (79.6±14.4 vs. 83.0±15.0, p=0.021),  Higher TG in Tertile 3 vs Tertile 1 of uPDI  (101.5±56.6 vs 97.7±56.5) |

hPDI=healthful plant-based diet index, uPDI=unhealthful plant-based diet index, PDI=plant-based diet index, FFQ=Food Frequency Questionnaire, T2D=Type 2 Diabetes, WC=Waist Circumference, FBS=Fasting blood sugar, GRS=Genetic Risk Score, HPFS= Health Professional Follow-up Study, CCHS= Canadian Community Health Survey, UK= United Kingdom, FI= Finland, NZ= New Zealand, BG= Bulgaria, AU= Australia, US= United States, na= not available

Table S5: Overview over studies assessing the association between the plant-based diet index and mortality

| Study | | | Population Characteristics | | | PDI | | | |  |
| --- | --- | --- | --- | --- | --- | --- | --- | --- | --- | --- |
| Authors | Outcome, assessment method | Study Design | n (% female) | Age in years | Country | Version | Dietary assessment | Mean±SD,  Median [range],  Median quantile 1-x | Energy-adjustment | Main Findings |
| Kim, 2019 [25] | CVD- and all-cause mortality  n_deaths_=5436  n_CVDdeaths_=1565,  self-reported, hospital records | Prospective | 12,168 (55.9%) | 53.8±5.7 | US | - Revised PDI. hPDI, uPDI - 17 food groups - Range 17-85 | FFQ | Q1-5  PDI 47-66  hPDI 29-61  uPDI 43-59 | Residual method,  Covariate | HR_all-cause mortality_=0.75 (0.69, 0.82) for PDI and 0.89 (0.8, 0.98) for hPDI,  HR_CVD-mortality_=0.81 (0.68, 0.97) for PDI and 0.68 (0.58, 0.80) for hPDI |
| Lazarova, 2022 [27] | Cardiovascular disease (CCHS 2004, n_events_=748),  Hospital and death records | Cross-sectional | CCHS 2004: 6771 (n.a.) | n.a. | Canada | - Revised hPDI, uPDI and PDI - 18 food groups - Range 18-90 | 24h diet recall | PDI 39.11± 0.14  hPDI: 43.2± 0.16  uPDI: 41.8± 0.16 | Covariate | No significant association with CVD risk |
| Thompson, 2023 [30] | Mortality  n_deaths_=5627  n_CVDdeaths_=698,  medical and death records | Prospective | 126,394 (55.9%) | 56.1±7.8 | UK | - Revised hPDI and uPDI - 17 food groups | 24h diet recall | Q1-4  hPDI: 47.7-63.4  uPDI: 46.6-61.8 | Covariate | HR_all-cause mortality_ =0.84 (0.78, 0.91),  comparing extreme quartiles of hPDI,  HR_all-cause mortality_ =1.23 (1.14, 1.32) comparing extreme quartiles of uPDI |
| Weston, 2022 [31] | All-cause mortality  n_deaths_=597,  Self-reported, hospital records | Prospective | 3635 (64.3%) | 53.8±12.5 | US | - Revised PDI, hPDI, uPDI - 18 food groups - Range 18-90 | FFQ | Q1-3  PDI: 48-61  hPDI: 48-60  uPDI: 48-61 | Residual method | No significant associations |
| Baden, 2019 [68] | Total mortality  n_deaths_=17,176  Cause-specific mortality  n_CVDdeaths_=3918,  death records, family reports | Prospective | NHS 49,407 (100%)  HPFS 25,907 (0%) | NHS 63.7  HPFS 62.9 | US | - Original hPDI, uPDI and PDI - 18 food groups - Range 18-90 | FFQ | Q1-5  PDI: 50.0-59.0,  hPDI: 49.0-60.0  uPDI: 49.0-59.0 | Covariate | HR_all-cause mortality_=0.95 (0.90,1.00) for PDI, 0.90 (0.85,0.95) for hPDI and 1.12 (1.07,1.18) for uPDI, comparing greatest increase vs stable diet scores  HR_CVD-mortality_=0.93 (0.88, 0.99) for PDI, 0.91 (0.86, 0.96) for hPDI and 1.08 (1.02, 1.14) for uPDI per 10-point increase in diet index |
| Delgado-Velandia, 2022 [69] | All-cause mortality  n_deaths_=699  CVD mortality  n_CVDdeaths_=157,  death records | Prospective | 11,825 (54.4%) | 47.0±0.3 | Spain | - Original hPDI, uPDI - 18 food groups - Range 18-90 | Diet history | hPDI: 56.3±0.1 uPDI:  56.2±0.8 | Covariate | HR_all-cause mortality_ =0.86 (0.74, 0.99) and HR_CVD-mortality_=0.63 (0.46,0.85) per 10-unit increase in hPDI |
| Kim, 2018 [70] | Total mortality  n_deaths_=2228,  death records  Cause-specific mortality  n_CVDdeaths_=543 | Prospective | 11,879 (54%) | 41.3±0.6 | US | - Revised hPDI, uPDI - 17 food groups - Range 17-85 | FFQ | Median [10^th^-90^th^ percentile]  PDI: 49 [43-58]  hPDI: 51 [43-60]  uPDI: 52 [44-61] | Covariate | HR_all-cause mortality_=0.95 (0.91, 0.98) per 10-unit increase in hPDI only in those with hPDI above median |
| Kim, 2021 [71] | Total mortality  n_deaths_=3074  Cause-specific mortality  n_CVDdeaths_=447,  death records | Prospective | 118,577 (65.1%) | 52.7±8.2 | South Korea | - Revised PDI, hPDI, uPDI - 17 food groups - Range 17-85 | FFQ | Q1-5PDI  PDI: 44-58  hPDI: 43-60  uPDI: 44-62 | Covariate, adjustment of food groups | HR_all-cause mortality_=0.76 (0.68, 0.85) for extreme quintiles of PDI  HR_all-cause mortality_=1.30 (1.15,1.48) for uPDI  HR_CVD-mortality_=1.55 (1.08, 2.25) for extreme quintiles of uPDI |
| Li, 2021 [72] | Total mortality  n_deaths_=4904  Cause-specific mortality  n_CVDdeaths_=1029,  death records | Prospective | 40,074 (52%) | 47.3±19.4 | US | - original hPDI, uPDI - 18 food groups - Range 18-90 | 24h diet recall | Q1-5PDI  PDI: 46-60  hPDI: 46-61  uPDI: 46-62 | Covariate | HR_all-cause mortality_ =0.80 (0.73,0.89) for extreme quintiles of PDI and 0.86 (0.77, 0.95) for hPDI and 1.33 (1.19, 1.48) for uPDI  HR_CVD-mortality_=1.42 (1.12, 1.79) for uPDI |
| Ratjen, 2021 [73] | All-cause mortality  n_deaths_=204,  death records | Prospective | 1404 (44%) | 69 [64-73] | Germany | - original PDI, hPDI, uPDI - 18 food groups - Range 18-90 | FFQ | Median (IQR)  PDI: 54 (50–58)  hPDI: 54 (49–59)  uPDI: 54 (49–59) | Covariate | HR_all-cause mortality_ =0.72 (0.57, 0.91) for PDI |
| Shan, 2023 [74] | Total mortality  n_deaths_=22,900  Cause-specific  n_CVDdeaths_=6641,  death records | Prospective | NHS: 75,230 (100%)  HPFS: 44,085 (0%) | NHS: 50.2±7.2  HPFS: 53.3±9.6 | US | - original hPDI - 18 food groups - Range 18-90 | FFQ | Q1-5PDI  44.6 ±3.2-64.6±3.3 | Covariate | HR_all-cause mortality_ =0.86 (0.83, 0.89) comparing extreme quintiles of hPDI  HR_CVDmortality_ =0.94 (0.89, 0.99) per 25 percentile increase in hPDI |
| Wang, 2023 [75] | Total mortality  n_deaths_=31,136  Cause-specific  n_CVDdeaths_=9751,  death records | Prospective | 315,919 (8.1%) | 65.5 (na) | US | - revised hPDI - 16 food groups - Range 16-80 | SFFQ | Q1-10PDI  PDI: 37.6-59.3  hPDI: 36.3-59.6  uPDI: 35.4-61.6 | Covariate | HR_all-cause mortality_ =0.75 (0.71, 0.79) for PDI, 0.64 (0.61, 0.68) for hPDI and 1.41 (1.33, 1.49) for uPDI comparing extreme deciles  Similar significant associations for CVD mortality |

hPDI=healthful plant-based diet index, uPDI=unhealthful plant-based diet index, PDI=plant-based diet index, FFQ=Food Frequency Questionnaire, CVD=Cardiovascular disease, NHS= Nurse’s Health Study, HPFS= Health Professional Follow-up Study, CCHS= Canadian Community Health Survey, UK= United Kingdom, US= United States, na= not available

Table S6 Overview over studies assessing the association between the plant-based diet index, cognitive impairment and gut microbiome

| Study | | | Population Characteristics | | | PDI | | | |  |
| --- | --- | --- | --- | --- | --- | --- | --- | --- | --- | --- |
| Authors | Outcome, assessment method | Study Design | n (% female) | Age in years | Country | Version | Dietary assessment | Mean±SD  Median [range],  Median quantile 1-x | Energy-adjustment | Main Findings |
| Baden, 2020 [76] | Health-related quality of life,  Self-reported | Prospective | NHS: 50,290 (100%)  NHSII: 51,784 (100%) | NHS: 58±7  NHSII: 39±5 | US | - Original hPDI, uPDI and PDI - 18 food groups - Range 18-90 | FFQ | Q1-5  PDI: 48.4±5.7 -60.9±5.7 | Covariate | Per 10-unit higher hPDI  β_PCS_=0.13 (0.08, 0.19)  β_MCS_=0.09 (0.03, 0.15)  Per 10-unit higher uPDI  β_PCS_=-0.07 (-0.12, -0.02)  β_MCS_=-0.10 (-0.16, -0.05)  Positive association of hPDI with PCS was significant among older females, and with MCS in younger females |
| Liang, 2022 [77] | Cognitive impairment  n_cases_=1077, MMSE | Prospective | 4792 (49.4%) | 80.7±9.6 | China | - Revised hPDI, uPDI - 16 food groups - Range 16-80 | FFQ | PDI: 48.71 ± 6.05, hPDI: 54.09 ± 5.38, and uPDI: 42.78 ± 6.65 | NA | HR_CI_=1.32 (1.16-1.50) for lower PDI, 1.46 (1.29, 1.66) for lower hPDI and 1.21 (1.06, 1.38) for higher uPDI  Protective effect of overweight was stronger among those with higher PDI (0.74 (0.57, 0.95)) and higher hPDI (0.73, 0.57, 0.94)) and lower uPDI (0.61 (0.46, 0.80)) compared to lower adherence |
| Liu, 2022 [78] | Cognitive decline,  MMSE, cognitive testing | Prospective | 3337 (64.0%) | 73.7±5.7 | US | - Original hPDI, uPDI - 18 food groups - Range 18-90 | FFQ | PDI: 53.3 ± 6.31, hPDI: 51.4 ± 6.78, and uPDI: 54.9 ± 6.72 | Covariate | β_globalCF_=0.0183±0.009, β_perceptualspeed_=0.0179 ±0.009 and β_episodicmemory_=0.0163±0.012 comparing extreme quintile of hPDI in African-american participants |
| Ma, 2023 [79] | Mood | Cross-sectional | 333 (66.1%) | 40.6±19.9 | UK | - Original PDI, hPDI, uPDI - 18 food groups - Range 18-90 | FFQ | PDI: 50.6 ± 6.4, hPDI: 51.6 ± 8.5, and uPDI: 51.6 ± 6.9 | NA | β_mood_=0.663, p=0.003 for PDI only in children |
| Van Soest, 2023 [80] | Cogntive ageing,  Cognitive testing battery | Longitudinal | 658 (41%) | 72.1±5.4 | Netherlands | - Original hPDI, uPDI - 18 food groups - Range 18-90 | FFQ | PDI: 54.0 ± 6.3 | Residual method | No significant association between PDIs and cognitive ageing  Potential interaction with fish consumption: β_globalCF_=0.12 (0.03, 0.21) per 10-unit increment of PDI only for individuals with high fish consumption |
| Wu, 2019 [81] | Cognitive impairment  n_cases_=2443,  MMSE | Prospective | 16,948 (59.2%) | 73.2±6.2 | Singapore | - revised PDI, hPDI - 15 food groups - Range 15-75 | FFQ | PDI: 39.8 ± 5.8  hPDI: 45.4 ± 5.6 | Covariate | OR_CI_=0.82 (0.71, 0.94) for PDI and 0.78 (0.68, 0.90) comparing extreme quartiles |
| Zhou, 2020 [82] | Healthy ageing  n_cases_=2834,  self-reported | Prospective | 14,159 (59.0%) | 53.3±6.1 | Singapore | - revised hPDI, PDI - 15 food groups - Range 15-75 | FFQ | Q1-5  PDI: 34-47, hPDI 39-52 | Covariate | OR_healthyageing_=1.34 (1.18, 1.53) for PDI and 1.45 (1.27, 1.65) for hPDI  OR_CI_=1.23 (1.06,1.43) for hPDI |
| Zhu, 2022 [83] | Cognitive function,  MMSE | Prospective | 6136 (46.3%) | 79.5±9.8 | China | - revised hPDI - 16 food groups, - Range 16-80 | SFFQ | PDI: 49.23±6.07  hPDI: 46.72±5.60  uPDI: 50.88±6.60 | NA | OR_CI_=0.45 (0.39, 0.52) for PDI, 0.61 (0.54, 0.70) for hPDI and 2.03 (1.79, 2.31) for uPDI comparing extreme quartiles |
| Hamaya, 2020 [86] | Circulating TMAO levels,  Blood measurements | Prospective | 620 (0%) | 67.7±7.7 | US | - Original PDI. hPDI, uPDI - 18 food groups - Range 18-90 | FFQ  7DDR | PDI 53[49-57]  hPDI 53 [49-58]  54 [49-59] | Covariate | β_TMAO_= 0.0015 (0.0007,0.023) for hPDI, -0.013 (-0.021, -0.005) for uPDI |
| Heianza, 2020 [87] | Circulating TMAO levels and CHD incidence  n_cases_=380,  medical records, blood measurement | Prospective case-control | 760 (100%) | 58.2±6.5 | US | - Original hPDI - 18 food groups - Range 18-90 | FFQ | NA | Covariate | RR_CHD_= 1.33 (1.06, 1.67) per 1 SD increment TMAO, this association was attenuated by hPDI adherence: RR_CHD_=1.48 for low adherence vs. 1.25 for high adherence per 1 SD increment of TMAO |
| Liu, 2021 [88] | Gut microbiota metabolites CAD risk  n_cases_=608,  medical records, blood measurements | Prospective case-control | NHSII: 374 (100%)  HPFS: 842 (0%) | NHSII: 45.7±4.1  HPFS: 63.6+8.7 | US | - Revised hPDI - 17 food groups - Range 17-85 | FFQ | Cases: 54.6 ± 7.4  Controls:  55.3 ± 7.2 | NA | OR_CAD_= 0.58 (0.38, 0.90) for high enterolactone/low TMAO profile 🡪 participants with this profile had significantly higher hPDI scores (56.0 (55.1, 56.8) compared to those with low enterolactone/high TMAO profile (54.1 (53.3, 54.8)) |
| Miao, 2022 [89] | Gut microbiome composition,  Fecal samples | Prospective | 3096 (52.3%) | 51.5+12.5 | China | - Revised PDI,hPDI, uPDI - 15 food groups - Range 15-75 | FFQ, 24-h diet recall | PDI: ,  45±5 | Covariate | Higher short-term hPDI associated with higher Shannon’s diversity index and Pielou’s evenness (β=0.15 and ≈0.20 respectively),  Higher long-term PDI associated with lower abundance of Firmicutes (Q5 vs Q1 β=-0.15 (-0.26, -0.03))  Four gut microbial features of long-term plant diet associated with HDL-C, LDL-C, TG and CRP (q<0.25) |

hPDI=healthful plant-based diet index, uPDI=unhealthful plant-based diet index, PDI=plant-based diet index, NHS= Nurse’s Health Study, HPFS= Health Professional Follow-up Study, UK= United Kingdom, US= United States, na= not available

7DDR: seven day dietary record, FFQ=Food Frequency Questionnaire, TMAO= trimethylamine N-oxide, CI=Cognitive impairment, CAD=coronary artery disease, CHD=coronary heart disease,MMSE=Mini Mental State Examination
